# Management of the COVID-19 pandemic: challenges, practices, and organizational support

**DOI:** 10.1186/s12912-022-00972-5

**Published:** 2022-07-22

**Authors:** Eman Kamel Hossny, Sahar Mohamed Morsy, Asmaa Mohamed Ahmed, Manal Saleh Moustafa Saleh, Atallah Alenezi, Marwa Samir Sorour

**Affiliations:** 1 Nursing Administration, Faculty of Nursing, Assiut University, Assiut, Egypt; 2 Nursing Administration, Faculty of Nursing, South Valley University, Qena, Egypt; 3 Nursing Administration, Faculty of Nursing, Zagazig University, Sharkia, Egypt; 4 Nursing Department, College of Applied Medical Sciences, Shaqra University, Shaqra, Saudi Arabia; 5 Nursing Administration, Faculty of Nursing, Tanta University, Tanta, Egypt

**Keywords:** Challenges, Practices, Organizational support, Nursing managers, COVID-19 pandemic

## Abstract

**Background:**

Health organizations currently face tremendous challenges in the management of the COVID-19 pandemic. To do this, successful and proven scientific practices and support are needed.

**Aim:**

This study aimed to explore the challenges, practices, and organizational support dealt with by nursing managers in the management of the COVID-19 pandemic.

**Method:**

A qualitative content analysis study evaluated 35 nursing managers in five university hospitals through a semi-structured interview. The Consolidated Criteria for Reporting Qualitative Research were used for this qualitative study.

**Results:**

Three main themes emerged: Challenges include the development of a COVID-19 crisis management plan, a shortage in nursing staff, and psychological problems. Practices include; changes in work schedules for nursing staff, the exchange process, hospital preparation, and training and education. And organizational support includes both support at an organizational level and support at an individual level.

**Conclusion:**

This study revealed that nursing managers are faced with many challenges in the management of COVID-19, requiring good practices and organizational support. This study offers evidence for nursing managers to expect problems that may arise during the pandemic.

**Recommendations:**

The COVID-19 pandemic requires the development of an integrated plan, and this plan must be disseminated to the hospital’s nursing and medical teams to better equip them for the current and future crises.

## Background

The COVID-19 pandemic started in Wuhan, China, in December 2019 and has spread intensely around the world since then, despite quarantine and containment measures [1].Sometimes crises are beyond human control and usually lead to death and affect people's lives in different ways. The crisis is a complex phenomenon that requires multi-directional corrective actions and, above all, preventive measures. There is no best way out of the crisis. Based on the facts of an absence of preparedness plans in hospitals, nursing managers face difficulties in managing the crisis during the outbreak of COVID-19, so studying the reality is the main target [2].

According to the World Health Organization (WHO), on April 13, 2019, more than 1.7 million people were infected, and nearly 85,000 people lost their lives. In addition, as of September 26, 2020, about 213 countries all around the world have been affected by this disease, resulting in 32,700,000 confirmed cases and more than 993,000 deaths.

In Egypt, the first positive case of COVID-19 was confirmed on February 14, 2020 [3]. Then there was a rise in the total number of cases and deaths. In parallel, there was a growing rise in the number of infected nurses and physicians [4]. In October 2020, Egypt was the 46^th^ country in the entire world and the 8^th^ Arab country affected by this virus. The number of cases has reached more than 106,707, and the number of deaths has reached more than 6,211. Moreover, this statistic is changing every day.

The COVID-19 pandemic is an extraordinary international health “war” in which the arena is the hospitals and our fighters are the health workers on the front lines. COVID-19 has presented nurses with unprecedented professional, social, and psychological challenges [5, 6]. Therefore, proper preparation of nurses is essential, as they are the greatest providers of health information and services for people [7]. The responsibility of this preparation falls on the nursing managers. Managers must be prepared to respond to the effects of this pandemic on themselves and their staff. Even with advances in healthcare and virus control technology, real success is not possible without effective leadership.

Nursing managers in health organizations currently face huge challenges related to dealing with pandemic operations. Therefore, managing the coronavirus crisis requires successful and proven scientific practices implemented through effective institutional work. Effective institutional work refers to the complete disappearance of vision and personal effort; the existence of institutions and entities that integrate with one another according to an interlocking system and a clear vision; the existence of methodologies and scientific foundations; the presence of a working group of people competent in the three levels of crisis management (strategic, executive, and operational); coordination among institutions, the optimal use of resources through a professional operating system that applies best practices; and the presence of many capabilities and local enablers that can be managed and built quickly and efficiently [8].

The scenarios for facing each pandemic wave need greater preparation and considerable experience in identifying the challenges, scientific practices, and organizational support. Since the beginning of the pandemic, the Egyptian Ministry of Health (MOH) has been using hotlines and sponsored advertisements on Facebook to teach people about the disease as a way to use such powerful tools to its advantage [9].

Also, leaders and nursing managers can provide specific support to their healthcare organizations and develop programs to cope with the coronavirus pandemic. This study can guide nursing managers in this regard [10, 11]. A coordinated global response is needed to prepare healthcare systems to face these challenges [12]. The World Bank emphasizes that global readiness for pandemics is crucial for global security and should be considered part of a program for strengthening healthcare systems [13]. Therefore, the researchers in this study sought to explore nursing managers’ experiences of the challenges, practices, and organizational support during the management of the COVID-19 pandemic. The study's findings would be useful to public health administrators and policymakers in determining how to manage crisis waves effectively.

### Significance of the study

This study was conducted in Egypt after the first pandemic wave in March 2020, for which more than 194 million COVID-19 cases have been reported in over 188 countries. Egypt was no exception and recorded the highest rate of coronavirus infections at 1,774 cases in June. The Ministry of Health recorded the highest death rate from COVID-19 with 97 deaths on June 15, 2020. At the beginning of the second wave in November 2020, the daily infection rate reached 365 recorded cases, but it jumped to 911 cases within days, with 42 deaths. As the world faces an unprecedented threat, an opportunity to achieve stronger healthcare systems and improve global cooperation is presented to confront the next health threat and enhance future pandemic preparedness [14].

Crisis management is the biggest challenge facing healthcare organizations. A good manager should consider the latest knowledge and practices, not only official ones, but also practical skills and experience in developing and implementing corrective agendas for crisis situation management. Significant knowledge gaps still need to be filled through ongoing monitoring and research activities. In the management of the coronavirus crisis, officials are exposed to many challenges that require serious consideration and solidarity among all responsible authorities to overcome obstacles traditionally inherent between nurses and doctors [15], and in healthcare and social sciences [16]. Determine the appropriate practices and support needed to reach and achieve the desired COVID-19 pandemic management goals. To this end, a qualitative approach was adopted because it is based on the participants’ first-hand experiences and yields more realistic results [17].

### Aims of the study

To explore the challenges, practices, and organizational support facing nursing in the management of the COVID-19 pandemic.

### Research objectives

1. Determine the challenges faced by nursing managers in the management of the COVID-19 pandemic.
2. Determine which common practices are being used in managing the COVID-19 pandemic.
3. Determine the types of organizational support provided in managing the COVID-19 pandemic.

### Study questions

1. What are the challenges nursing managers face in managing the COVID-19 pandemic?
2. What are the practices being used to face the challenges of managing the COVID-19 pandemic?
3. What are the types of organizational support provided?

## Subject and methods

### Study design and setting

This qualitative study design was conducted in 2020 in five university hospitals in Egypt. This design was considered suitable for exploring the challenges, practices, and organizational support facing nursing managers in the management of the COVID-19 pandemic. It could also provide the participants with time and an opportunity to speak openly and reflect deeply on their personal experiences. All of these hospitals offer scientific learning and exercise to upcoming and existing physicians, nurses, and other healthcare personnel while providing therapeutic care to patients. These hospitals are collectively referred to in this study as universities.

### Study subject and data collection

The participants were enrolled (35 nurse managers), including five nursing directors, five assistant nursing directors, 10 supervisors, and 15 head nurses at five university hospitals in Egypt. All participants were female, with a mean age of 41.36 ± 6.66 years and a mean work experience of 19.10 ± 5.57 years. Among all the participants, three had PhDs, 18 had master’s degrees, and 14 individuals had bachelor’s degrees in nursing.

Sampling was based on a purposive sampling technique to get an in-depth understanding of the situation during the pandemic and was also used to select diverse participants from the five hospitals. This ensured that the participants with diverse experiences clarified different aspects related to the aim of the study. The following inclusion criteria were applied: being at one of the nursing management levels, having worked in it for a period of no less than two years, and having more than five years of experience.

The researchers communicated directly with the nursing directors by phone and also sent an e-mail to inform them about the study. The researchers conducted semi-structured face-to-face and telephone interviews to collect data [18]. This study was conducted after the first wave of the COVID-19 pandemic, which began in September 2020 and was completed in November 2020, (total time frame for data collection of about 5 weeks).

The researchers used open-ended questions to encourage discussion with the interviewees and obtain more in-depth information. The interview encompassed nursing managers’ challenges, practices, and organizational support in managing the COVID-19 pandemic. The researchers used the following questions as a guide in the interview: What challenges have nursing managers faced during the COVID-19 pandemic? Is there any pre-preparation for this crisis? What practices are used to overcome the problems raised? Can nursing managers elaborate on these practices? What types of organizational support were provided during the COVID-19 pandemic? The researchers conducted interviews until sufficient data for analysis was obtained. The 30-to 90-min interviews were conducted at the times and places selected by the contributors.

Operational definition for nurse manager: in the current study, nurse manager means one of those at the administrative level, either director/ assistant director, or supervisor, or head nurse.

### Data analysis

Two authors conducted semi-structured interviews to collect data. They are faculty members of the faculty of nursing and have previous experience as nursing managers in different hospitals. They do not work at either of the hospitals where participants were enrolled, which ensured that what the participants said in the interviews was not affected by personal relations with the two authors. All authors agreed on the final categories. During the interviews, the two authors’ interpretations of what the participants shared were validated by summarizing and asking questions about what they said to ensure that there were no misunderstandings. Credibility was enhanced by purposeful sampling from five different university hospitals.

The researchers used a traditional (conventional) content analysis approach, using Graneheim and Lundman’s method [19]. In this method, each entire interview was considered an analysis unit. The unit of analysis refers to the notes that must be analyzed and coded. The recorded interviews were replayed several times and transcribed accurately by the researchers. Paragraphs, phrases, and words that are linked to each other in terms of content are considered meaning units. They are classified according to their content and background. Written transcripts were revised numerous times to highlight words covering units of meaning and extract initial symbols. The codes were then reviewed numerous times in a continuous process from code drawing to classification. The same symbols were combined, classified, and named, and subdivisions were obtained. Finally, the extracted subcategories were compared and combined to form the major categories or themes. The researchers evaluated the data using the Consolidated Criteria for Reporting Qualitative Research (COREQ) [20].

### Assessment of data stability and accuracy

The stability and accuracy of data were checked using Lincoln and Guba’s criteria [21]. The believability of the data was evaluated using the triangulation method, member-checking, prolonged engagement techniques, and an external checking process (external researcher). The external evaluator is a member of the nursing administration with more than 20 years of experience, who reviewed the data after the semi-structured interview to ensure credibility.

## Results

Built on the outcomes of the interviews with nursing managers, the following three main themes and eight subthemes were related to “challenges,” “practices,” and “organizational support.” Challenges include development of a COVID-19 crisis management plan; shortage of nursing staff; and psychological problems. Practices included changes in work schedules for nursing staff, the exchange process (deal between university hospitals), hospital preparation, and training and education. Organizational support includes support at the organizational level and support at the individual level (Tables 1, 2, and 3).

**Table 1:** Categories, subcategories, and codes related to challenges in the management of the COVID-19 pandemic

Categories	Subcategories	Open codes
***Development of a* COVID-19 *crisis management plan***	Ad hoc committee	- (100%) of top-level nursing managers asserted that no preparedness plan had been in place - An Ad hoc committee was formed at the university hospitals’ level - A committee was assigned with the tasks and responsibilities to establish a preparedness plan for confronting the COVID-19 pandemic - A committee was composed of different department managers of the university hospitals
	Use the plan of another isolation hospitals (*use Aboteage Hospital’s and Esna Hospital’s plans)*	*-* Use the plan of another isolated hospital as a benchmark on how the plan will respond to the crisis
***Shortage in nursing staff***	Absenteeism among nurses	- Fear of infection makes nurses use their vacation leaves to be absent - Curfew makes (night transportation difficult for remote villages during curfew, reflecting negatively on night shift nurses)
	Infections among nurses	- Exposure - Work with suspected and positive cases
***Psychological problems***	-Panic emotions, fear, anxiety, and depression -Emotional motivation through good relationships between nursing managers and their nurses -Physically self-existence of nursing managers with nurses -Provision of psychological cession to nurses	-Related to the nature of the coronavirus and fear of infection, dealing with suspected cases, and caring for COVID-19 patients -Insufficient nursing knowledge about the coronavirus -Refuse to deal with nurses from the community -Cut family ties with nurses

**Table 2:** Categories, subcategories, and codes related to practices in the management of the COVID-19 pandemic

Categories	Subcategories	Open codes
***Changes in work schedules for nursing staff***	In regular work places In isolation places	-Day and night shift only instead of three shifts, from 7AM–4 PM and from 4 PM–7 AM -Nurses stay 24 h; each nurse works 12 h a day and swaps shifts every 6 h
***Exchange process (deal between university hospitals)***	The deal in regular work places	- Elderly nurses, nurses who support their families, nurses who have children, as well as nurses who have chronic diseases, work in regular places
	The deal in isolation places	- young nurses who do not have spouses or children and are free from chronic diseases to work in isolation hospitals (one university hospital) and partial isolation places in each university hospital with many facilities, such as: - Full accommodation in university cities for 14 days - Provision of transportation - Transferring of signature to isolation facilities - Granting exceptional leave to workers in isolation - Exemption from signing for infected staff
***Hospital preparation***	Equipping the hospital	- The rapid-response team is appointed to deal with suspected and positive cases - Allocate rooms to sort the cases - Allocate rooms to isolate positive cases - Equipping ambulances by providing hand sanitizers, pumps for spraying chlorine, and personal coasters for the driver and paramedic - The morgue; training of workers on PPE use, disinfection, and sterilization, and posters on how to deal with COVID-19 deaths and mortuary refrigerators
	Infection control team	-Liaison between the Supreme Council of Universities and hospital managers -Monitoring to ensure medical and nursing teams and workers are wearing the personal protective equipment (PPE) correctly and in the appropriate places -Preparing reports about suspected cases and infected cases and the whole plan related to their role - The allocation of a tripartite committee to monitor the disbursement of personal protective equipment - Provide temperature detection reagents
***Training and education***	Training	- Conducting intensive training for the medical team - Training for doctors on performing swabs - Training on how to wear and remove PPE - Training on how to deal with suspected and infected cases
	Education	- Small lectures - Small education teams were assembled to teach doctors, nurses, and workers how to deal with each other and with suspected and infected cases

**Table 3:** Categories, subcategories, and codes related to organizational support in the management of the COVID-19 pandemic

Categories	Subcategories	Open codes
***Support at organizational level***	Determining the paths and places of isolation in each hospital	-A separator was put between isolation rooms and administrative offices -The allocation of triage and isolation rooms for suspected patients in the general reception (no = 21)
	Allocate Assiut university isolation hospital	1-Isolation hospitals serve faculty members and employees at the university 2- It was divided into three zones according to the condition of the patients; - Green zone, it receives positive cases that have no symptoms or have simple symptoms - Yellow zone, it receives positive cases that have severe symptoms, but do not need respirators - Red zone, it receives positive cases that have severe symptoms, and need respirators 3-It was divided into two areas according to the possibility of contamination; -The clean area includes restrooms, an elevator and a staircase -The contaminated area includes patient rooms and patient ward elevator, where transportation of patients, samples, and contaminated laundry were done 4-Separations have been made to isolate between the clean area where contaminated area
***Support at individual level***	Communication and collaboration	- Nurses and physicians collaborate in providing care to patients during hospitalization, - Physicians provided medical information to nurses when needed, which was facilitated by what Sapp group to facilitate communication between health care teams - Open phones for in charge personnel 24 h a day for both medical and nursing teams
	Material support	-The Supreme Council of Universities provided the university hospitals with antiseptics and preventive supplies - Provision of financial rewards to all medical staff -Provision of antiseptics and preventive materials to the hospital medical team -Transportation provision -Provision of cameras in isolation facilities -Transferring of signatures in isolation facilities, granting exceptional leave to workers in isolation and those with chronic diseases, and exemption from signing for infected staff -Transferring of signatures to isolation facilities, granting exceptional leave to workers in isolation and those with chronic diseases, and exemption from signing for infected staff

### Theme 1: Challenges (Table 1)

Nursing managers while managing the COVID-19 pandemic face many challenges that need creative thinking to solve. Semi-structured interviews revealed three sub-themes: development of a COVID-19 crisis management plan; shortage of nursing staff; and psychological problems.

### Development of a Covid-19 crisis management plan

Most nursing managers confirmed that preparation for a COVID-19 crisis management plan was the biggest challenge they faced. The coordination, planning, and successful implementation of adaptable COVID-19 preparedness and response strategies will depend on all hospitals’ being engaged in the plan, as well as constant coordination. The factors related to this subtheme are discussed subsequently.

### Ad hoc* committee*

All five (100%) top-level nursing managers asserted that no preparedness plan had been in place. A committee was formed at the university hospitals level and composed of different department managers of the university hospitals, and was assigned the tasks and responsibilities of establishing a preparedness plan for confronting the COVID-19 pandemic. Nursing director no. 1 said, *“The committee was formed at the university hospitals level. The committee was composed of different department managers of the university hospitals (nursing, medical, pharmacist, laboratories, engineering, physicians with different specialists, and infection control).”*

*Use the plan of another isolation hospitals* (*use Aboteage Hospital’s and Esna Hospital’s).*

Hospital managers used the plan of another isolated hospital as a benchmark, to see if they were going in the right direction, in addition to different suggestions, if available. Nursing director No. 5 said, *“We use Aboteage Hospital’s and Esna Hospital’s plans to determine whether we are in the right direction.”*

### Shortage in nursing staff

All nursing managers agreed that the shortage of nursing staff was the second biggest challenge faced by all hospitals. Without a sufficient number of nurses, the healthcare sector cannot provide adequate care to patients. The factors related to this subtheme are described herein.

#### Absenteeism and presenteeism among nurses

The absenteeism rate among nurses increased because of many factors, such as fear of infection, curfew, psychological problems, remote villages, and difficult transportation. According to interviewees, curfew was considered the main cause of nurse absenteeism aside from fear of infection. Nursing managers in the main hospital said, “Curfew started at 5:00 pm in all counties, so night shift nurses can’t go to their workplace, especially since about 55% of them live in rural areas (remote villages). Besides the difficulty in getting transportation during curfew, nurse absenteeism from work is also due to vacation leaves.

Head nurse No.1 said, *“Three nurses in one unit were found positive for the coronavirus, which led to all the other nurses in the unit being absent and eventually causing this unit to be closed. In other words, fear of infection makes nurses use vacation leaves to be absent.”*

#### Infection among nurses

The coronavirus started to spread among nurses in different hospitals and units. Contact with infected people and work with suspected and infection cases were the main causes of this spread. More than half of first-line nursing managers said, that “most nurses use university buses for transportation, which contributes to the spread of infection among them.”

Supervisors’ No. 1, 2, 3, and 4 said, *“Nurses became infected from contact with other nurses in hospital transportation and from lending personal items, all of which contributed to the high number of infections among nurses.”*

### Psychological problems

Nurses’ experienced psychological problems during COVID-19 and fear of infection were the main factors related to this challenge.

#### Panic emotions, fear, anxiety, and depression

At the height of the pandemic, all healthcare personnel were distressed, and some of them became panicked about infection. Nursing directors asserted that nurses’ fears are legitimate. Also, related to the nature of the coronavirus, and fear of infection, dealing with suspected cases, and caring for COVID-19 patients, insufficient nursing knowledge about the coronavirus contributed to nurses’ fear of exposure to infection, making them susceptible to anxiety and worry. Moreover, bad interactions with family members, neighbors, and their community contribute to nurses’ psychological problems and depression.

Nursing directors’ No. 1, 2, 3, 4, and 5 said, *“Nurses are afraid of infection. One of the nurses said, I will not come to work; I fear becoming infected.”*

All the head nurses (no = 15) said, *“Nurses ask about the virus and how to protect themselves from infection.” They are also afraid for their children and family members.*

One head nurse said, *“Some nurses in my unit cry due to bad dealings with their husbands or their neighbors.”*

### ***Theme 2: Practices (Table ******2******)***

Nursing managers provided creative practices in crisis management throughout the COVID-19 pandemic. These practices included changes in nursing staff work schedules, the exchange process (a deal between university hospitals), hospital preparation, and training and education.

### Changes in work schedules of nursing staff

#### In regular workplaces

Nursing managers had to match nurses’ work hours with curfew hours. They had to change the work schedule to two shifts a day (morning and night), instead of three shifts (morning, evening, and night) which started from 7:00AM to 4:00 PM and from 4:00 PM to 7:00 AM, as attested by the directors of the hospitals.

Nursing director No. 1 said, *“We had to change the work schedules to cope with the curfew conditions in the country.”*

### In isolation places

The isolation facilities also needed their work hours to change to allow nurses on duty to work 6 h per shift, have a break in the next 6 h before working again in the next 6 h to work exactly 12 out of 24 h a day for 14 consecutive days. However, the nurses in isolation facilities have full accommodation, whereas the other nurses who are working in the usual work settings do not.

All assistant nursing directors under study (no = 5) said, *“In isolation facilities, nurses stay 24 h for 14 days with full accommodation, which necessitates an appropriate work schedule to cope with such conditions.”*

### Exchange process (deal between university hospitals)

One of the most important practices that were used to solve many problems during the COVID-19 pandemic crisis was the exchange process. This process took place at the level of the five university hospitals to meet the shortages of staff nurses. The deal provides for taking young nurses who do not have spouses or children to work in an isolation hospital (one university hospital) and partial isolation places in each university hospital, in exchange for giving elderly nurses, nurses who support their families, nurses who have children, as well as who have chronic diseases the opportunity to work in regular workplaces.

*"Deal between the university hospitals solves the problem of nursing shortage as well as the creativity in applying it in isolated places and regular places,"* said Nursing Director No. 5.

### Hospital preparation

Hospitals must be prepared and well furnished with various equipment and supplies to face the coronavirus pandemic. The factors related to this theme include equipping the hospital and infection control team.

#### Equipping the hospital

Equipping the hospital with a trained rapid-response team to deal with suspected cases, and separate sorting rooms for suspected and positive cases. Moreover, equipping ambulances and morgues with hand sanitizers, pumps for spraying chlorine, personal coasters for the driver and paramedic, and training on dealing with patients. The morgue was prepared as well, with staff training, posters on how to deal with the dead and mortuary refrigerators, and instructions on properly wearing PPE to prevent infection.

Supervisors’ No. 1, 3, and 4 said, *“Equipping hospitals well will help them deal with the pandemic and better organize the work flow through a trained rapid-response team to deal with suspected cases and separate sorting rooms for suspected and positive cases, as well as equip ambulances and morgues with needed supplies.”*

Head nurses No. 1, 2, and 3 said, *“We supervise infection control nurses and ensure that each car has enough protective measures and that the morgues are equipped and adequately sterilized.”*

#### Infection control team

All of the nursing managers interviewed asserted the significant role of an infection control team during the COVID-19 pandemic, in which they have a pioneering role in the liaison role between the Supreme Council of Universities and hospital managers for providing any instruction or information. They are also preparing reports about suspected cases and infected cases and the whole plan related to their role, in addition to monitoring medical and nursing teams to monitor their commitment to wear personal protective equipment (PPE) as well as monitoring the worker. Moreover, the appointment of a tripartite committee to monitor the disbursement of personal protective equipment (PPE) in addition to providing temperature detection reagents.

The nursing directors in each hospital said, *“The infection control unit plays a vital role in providing PPE and daily monitoring reports on all nurses, physicians, and hospital workers.”* They constitute a liaison between the Supreme Council of Universities and hospital managers.

### Training and education

Training and education are the backbones of the management of this pandemic. Adequate training and information on how to deal with COVID-19 need to be disseminated to all healthcare teams.

#### Training

Intensive training should be provided to the medical team (physicians, nurses, and other healthcare workers), such as on conducting swab tests for suspected cases. Furthermore, training on how to wear and remove PPE should be conducted in sorting and isolation areas.

One assistant nurse director said, *“Training started in March 2019 to equip the nurses, physicians, and workers to face the ongoing pandemic.”*

#### Education

Small education teams were prepared to educate all doctors, nurses, and other healthcare workers about COVID-19. Nurses in isolation rooms were also taught how to deal with infected patients without exposure to infection.

One director said, *“Education for all medical staff was conducted in addition to small lectures in all hospitals to teach nurses in isolation areas.”*

### Theme 3: Organizational support (Table 3)

In the management of the coronavirus crisis, support required to reach and achieve the desired goals in successful COVID-19 pandemic management. Themes related to this them were; support at organizational level and support at individual level.

### Support at organizational level

#### Determining the paths and places of isolation in each hospital

At the level of all university hospitals, a separator was made between isolation rooms and administrative offices. The allocation of triage and isolation rooms for suspected patients in each hospital.

#### One supervisor said that the general reception in the main hospital has triage and isolation rooms that reach to (no = 21)

### Allocate Assiut University’ isolation hospital

One of the organizational supports that helped nursing managers and medical managers manage the COVID-19 pandemic was the Allocate Assiut university isolation hospital. It was divided into three zones according to the condition of the patients (COVID-9 triage). Green zone, it receives positive cases that have no symptoms or have simple symptoms, yellow zone, it receives positive cases that have severe symptoms, but do not need respirators, and red zone, it receives positive cases that have severe symptoms, and need respirators. The hospital was divided into two sections based on the risk of contamination: the clean area, which includes restrooms, an elevator, and a staircase, and the contaminated area, which includes patient rooms and a patient ward elevator that transports patients, samples, contaminated laundry, and so on. *Separations have been made to separate the clean and contaminated areas.*

Nursing Director No. 5 said*, “Faculty and university employees are served by the isolation hospital. It was divided into three zones according to the condition of the patients (green zone, yellow zone, and red zone). It was also divided into two areas according to the possibility of contamination (clean area and contaminated areas). Moreover, Separations have been made between the clean area and the contaminated area.”*

### Support at an individual level

Managerial support for health care providers was inevitable, especially at a time of crisis. Subcategories related to support at an individual level included communication and collaboration, and material support.

#### Communication and collaboration

Communication and collaboration among the five hospitals are also counted as organizational support at an individual level between healthcare professionals. The main features include: nurses and physicians collaborate in providing care to patients during hospitalization; physicians provide medical information to nurses when needed; what Sapp group to facilitate communication between health care teams; and open phones for in-charge personnel 24 h a day for both medical and nursing teams.

One director said, *“The communication and collaboration between medical staff and nursing staff on all managerial levels has been unprecedented in the history of nursing, with nurses and physicians working together as one team in regular and isolated places.”*

One supervisor said*, “What Sapp group was initiated to facilitate communication between us, staff nurses, medical staff, as well as nursing and medical managers, in addition to open phones 24 h a day for those in managerial positions.*

#### Material support

The Supreme Council of Universities provided antiseptics and preventive supplies to university hospitals; financial rewards to all nurses and doctors who work in isolation facilities; cameras in isolation hospitals; signature transfer to isolation facilities; exceptional leave for workers in isolation and those with chronic diseases; and exemption from signing for infected staff.

All five nursing directors reported that “*The Supreme Council of Universities provided the university hospitals with antiseptics and preventive supplies needed during the pandemic. In addition to the provision of financial rewards to all medical staff (nurses and physicians who work at isolation places), and the provision of nutrition and treatment. Furthermore, all floors in the isolation hospital have been installed with cameras to follow up work, monitor nurses and doctors, and support actions for healthcare personnel and patients, transferring signatures to isolation facilities, granting exceptional leave to workers in isolation and those with chronic diseases, and exempting infected staff from signing.”*

## Discussion

This study was conducted through interviews with a total of 35 female nursing managers with a mean age of 41.36 ± 6.66 years and a mean work experience of 19.10 ± 5.57 years, including five nursing directors, five assistant nursing directors, 10 supervisors, and 15 head nurses, aiming to explore the challenges, practices, and organizational support dealt with by nursing managers in the management of the COVID-19 pandemic.

In this study, in Table 1, managers reported that the biggest challenge they faced at the start of the pandemic was the development of a plan to deal with the COVID-19 pandemic. According to those interviewed in his study, there might have been no plan that helped deal with the COVID-19 pandemic had been in place, but hospitals are usually required to have a crisis management or emergency response plan. The study conducted by Mathew et al., [22] found that lack of pandemic preparedness plan was one of the challenges faced by frontline health and social care workers during COVID-19 pandemic.

So as revealed from the interviews all activities related to the preparation were internally initiated. An Ad hoc committee was formed at the university hospitals level, composed of different department managers from nursing, medical, pharmacy, laboratories, engineering, physicians with different specialists, and infection control, assigned with the tasks and responsibilities to establish a preparedness plan for confronting the COVID-19 pandemic. These coordinated activities, what made the plan evolve in time. In many countries, strong coordination techniques were needed, as the fragmentation of health services resulted in inadequate responses and timely interventions to health emergencies [23].

The second biggest challenge facing nursing managers in this study was the shortage of nursing staff and figuring out how to work with 30% of hospital personnel. The research study by Matthew et al., [22] found several challenges faced by health and social care workers on the front lines during the COVID-19 pandemic, staff shortages, lack of personal protective equipment (PPE), and anxiety and fear among professionals. According to the result of the interviewees, the main reason for this shortage was absenteeism and fear of infection due to the lack of a clear plan and strategies for deal with the COVID-19 pandemic [24]. The WHO [25] recommends that staff shortages should be anticipated due to absenteeism and increased demand for services, and a plan should be put in place to address this shortage.

Regarding absenteeism, fear of infection was the main cause that make nurses use their vacation for absence. In addition, curfew makes transportation difficult especially in the night and in the remote villages, resulting in increase absenteeism in the night shift, putting nursing managers in a challenge. According to Adamczyk et al., [26] restrictions on movement and travel one of the challenges of the pandemic, In this study, nursing managers have been obliged to change the work schedules of the nursing staff to adapt to the curfew in Egypt (5:00 PM to 6:00 AM). The study by Partzick et al., [27] in Germany revealed that the COVID-19 pandemic has rapidly changed the working conditions of nurses and their working lives.

This change was imperative because, without the nurses, adequate care could not be provided to patients. In regular workplaces, the usual three-shift system (day, evening, and night) was revised to two shifts (9 h. a day and 15 h. a night), 7 AM–4 PM and 4 PM–7 AM. However, in isolation facilities, the changes in the work schedule involved implementing a four-shift day, with work for 6 h per shift and each nurse working two alternate shifts, and full accommodation provided (Table 2). This finding came in line with the policies applied in the region to overcome nurses’ shortage, as about 17 countries increased working hours and vacation time, in addition to implementing full-time work hours [28].

Moreover, to deal with the crisis, new and creative ideas were needed to solve the upcoming challenges innovatively. So, creativity and innovative solutions were needed to develop new working procedures and practices [29]. One of the most important and creative practices that were used to overcome shortages during the COVID-19 pandemic crisis was the exchange process (a deal between the university hospitals). The deal provides for taking young nurses who do not have spouses or children to work in isolated places, in return for giving elderly nurses, nurses who support their families, nurses who have children, and nurses who have chronic diseases the opportunity to work in regular workplaces (Table 2).

The third challenge facing nursing managers during the pandemic was psychological problems such as panic, fear, anxiety, and depression facing staff nurses. In many reported studies, staff nurses’ doubts regarding practices for carefulness with COVID-19 patients caused anxiety and fear. Furthermore, studies conducted in many countries related to caring for COVID-19 patients reported negative psychological consequences, such as anxiety, sadness, and stress, for nurses [30–33]. According to the study conducted in Egypt on health care workers (HCW) exposed to COVID -19, an extensive number of them had symptoms of anxiety, insomnia, depression, and stress [34]. Another study conducted on nurses in Egypt reveals that about three-quarters (75.2%) of nurses working in fever hospitals had high-stress levels versus 60.5% of nurses working in general hospitals [35].

From another prospective, the findings of a recent quantitative study on frontline nurses’ experiences showed that fear and unwillingness were mitigated through increased knowledge about the virus [36]. According to Adamczyk et al., [26] uncertainty about the future and there was a lot of contradictory information in the media caused a sense of confusion and heightened the feeling of anxiety. Dealing with the situation was not facilitated by the phenomenon of global misinformation, called by some experts as the “infodemic”, which may be defined as an overabundance of information that makes it difficult for people to find trustworthy sources and reliable guidance [37].

Both nursing managers and nursing staff also faced psychological problems. The COVID-19 outbreak causes panic emotions among nurses, and a lack of preparation to deal with these emotions puts them under great tension. However, nursing managers in our study, having a strong presence in their departments, being a good role models the staff, and acting as both leaders and administrator, have the authority to generate a more positive impact on their staff during this pandemic [38].

A study conducted by Gao et al. [39] found that a close relationship between nursing managers and their staff nurses was a vital factor in increasing motivation. However, perspectives on the significance of the psychological well-being of nurses during the COVID-19 pandemic should be emphasized [40]. Emotional motivation to relieve nurses from the stress they experience at work was demanded.

In addition to the role, that hospital preparations plays in fighting a COVID-19 pandemic, through equipping the hospital with a rapid-response team who are prepared to deal with suspected and positive cases, allocating rooms to sort the cases and others to isolate positive cases, and equipping ambulances and the morgue.

Furthermore, the role played by the infection control team at the level of all the studied hospitals is not neglected. From the researchers’ viewpoints, the infection control team was the backbones of each hospital which its team act as the Liaison between the Supreme Council of Universities and hospital managers, according to the WHO [5], in the face of an unknown disease, sharing and collaboration are the best means of finding solutions. According recent policy paper developed by Ghannam & Sebae [41] itemized amongst its commendations the need for improved infection control in health services in their attempt to study the effect of the pandemic on the new health insurance system that is being piloted in Egypt. The infection control team prepares reports about suspected cases and infected cases and the whole plan relato ted their role, a tripartite committee was created to monitor PPE disbursement [42]. PPE is one of the most important requirements for fighting an infectious pandemic like COVID-19. This is in addition to providing temperature detection reagents.

Furthermore, education and training play an important role in preparing nursing and medical staff to proficiently face this pandemic. According to the study conducted to assess the nurses' knowledge, concerns, perceived impact, and preparedness toward the COVID-19 pandemic, around half (51.2%) of the studied nurses said that the main sources of knowledge were from the Ministry of Health and World Health Organization's websites and formal pages [43]. It should be provided to all nursing and medical teams and healthcare workers. According to Chimenya [44], hospitals should conduct training and education sessions to bridge the knowledge gap and educate workers about their fear of infection.

In line with the overall findings of the practices presented in this study, several protective and preventive policies were emerged to overcome the negative effect of the pandemic, these policies included: management and communication, training and education, wellbeing drop-in sessions, peer support, team support, availability of personal protective equipment, and proper planning [45].

The third theme that will be discussed in this study is organizational support (Table 3). Organizational support is carried out at two levels, one of which is at the organizational level to determine the paths and places of isolation in each hospital and to allocate the isolation hospital at Assiut University to serve the university’s faculty and staff members, and which has been divided into three zones according to the condition of the patients (green, yellow, and red).

Another at the individual level was the evidence of communication and collaboration between nursing and medical management teams during the COVID-19 pandemic. Nurses and physicians need to know the importance of communication in healthcare [46]. Nurses and physicians collaborate in providing care to patients during hospitalization; regarding Hossny [47, 48], when nurses and physicians have the increased opportunity to work in a civil environment, they will collaborate more. Also, the establishment of information and communication channels was required during crisis management. Communication and collaboration in this study were demonstrated by the creation of WhatsApp groups and open phones for in-charge personnel 24 h a day for both medical and nursing teams.

Other ways of organizational support provided include material support, for instance, the Supreme Council of Universities provided disinfectants (antiseptics) and preventive supplies to all university hospitals, The university also provided transportation and full accommodation, financial rewards, transferring of signature in the isolation facilities, free swabs for all employees, and the granting of exceptional leaves to workers in isolation and those with chronic conditions, as well as the exemption from signature for infected staff, were implemented.

According to Hossny, [49] it is the responsibility of hospital managers to develop the weaker services inside their hospitals. This helps ourselves and our institutions prepare for this complex and fast economy, which is essential for support. Accordingly, many procedures related to obtaining signatures and facilities performed in the healthcare setting make work during the pandemic easier. Regarding signatures, all nursing managers reported that granting exceptional leave to workers in isolation and those with chronic diseases, as well as an exemption for infected staff from signing, was implemented. Regarding facilities, Open University cities for medical staff who want to stay in isolation and the provision of buses for nurses to facilitate transportation were established.

### Limitations of the work

About generalizing the results of this study, there are some restrictions, as the qualitative research is primary subjective in approach, which could carry some bias. This study was conducted in five university hospitals, while the university compound includes seven hospitals, also there are many hospitals affiliated with the Ministry of Health in Assiut city, in addition to health insurance hospitals. Furthermore, this study was restricted to nursing managers. Therefore, study various sectors of hospitals including all segments of staff nurses, will provide preferred informations.

## Conclusion

Nursing managers have gained much experience in crisis management throughout the COVID-19 pandemic. Nursing managers faced many challenges in the management of the COVID-19 pandemic. These challenges include developing a crisis preparedness plan, shortage of nursing staff, and psychological problems. Useful practices include adapting schedules, equipping and preparing hospitals, and training and education. In addition, organizational support is carried out at two levels, one of which is at organizational level to determine the paths and places of isolation in each hospital and to allocate the isolation hospital. Another at the individual level include establishing communication and cooperation, and material support such as providing disinfectants, financial rewards, and transferring the signature to the isolation facilities.

### Recommendations

The COVID-19 pandemic requires the development of an integrated plan, and this plan must be disseminated to the hospital’s nursing and medical teams to better equip them for the current and future crises. The importance of cooperation and effective communication should also be emphasized. Accurate implementation of instructions also helps overcome these crises and succeed in establishing and maintaining safety. More studies are required on how to manage the COVID-19 pandemic in hospitals.

## Data Availability

All data generated or analyzed during this research are included within this manuscript. The authors confirm that all methods were performed in accordance with the relevant guidelines and regulations.
